# Borna disease virus (BDV) circulating immunocomplex positivity in addicted patients in the Czech Republic: a prospective cohort analysis

**DOI:** 10.1186/1471-244X-10-70

**Published:** 2010-09-08

**Authors:** Sylva Rackova, Lubos Janu, Hana Kabickova

**Affiliations:** 1 Psychiatric Department, University Hospital, Medical Faculty Charles University in Pilsen, Alej svobody 80, Pilsen, 301 00, Czech Republic; 2 Klinlab s.r.o, Department of Molecular Biology and Parasitology, U Vojenské nemocnice 1200, Prague, 100 00, Czech Republic

## Abstract

**Background:**

Borna disease virus (BDV) is an RNA virus belonging to the family Bornaviridae. Borna disease virus is a neurotropic virus that causes changes in mood, behaviour and cognition. BDV causes persistent infection of the central nervous system. Immune changes lead to activation of infection. Alcohol and drug dependence are associated with immune impairment.

**Methods:**

We examined the seropositivity of BDV circulating immunocomplexes (CIC) in patients with alcohol and drug dependence and healthy individuals (blood donors). We examined 41 addicted patients for the presence of BDV CIC in the serum by ELISA at the beginning of detoxification, and after eight weeks of abstinence. This is the first such study performed in patients with alcohol and drug dependence.

**Results:**

BDV CIC positivity was detected in 36.59% of addicted patients on day 0 and in 42.86% on day 56. The control group was 37.3% positive. However, we did not detect higher BDV CIC positivity in addicted patients in comparison with blood donors (p = 0.179). The significantly higher level of BDV CIC was associated with lower levels of GGT (gamma glutamyl transferase) (p = 0.027) and approached statistical significance with the lower age of addicted patients (p = 0.064). We did not find any association between BDV CIC positivity and other anamnestic and demographic characteristics.

**Conclusions:**

In our study addicted patients did not have significantly higher levels of BDV CIC than the control group. The highest levels of BDV CIC were detected in patients with lower levels of GGT and a lower age.

**Trial registration:**

This study was approved by the ethical committee of the University Hospital Medical Faculty of Charles University in Pilsen, Czech Republic (registration number 303/2001).

## Background

Psychiatric disorders are a wide group of various diseases with heterogeneous etiologies (genetic predisposition, exposure to stress, environmental factors). It has been suggested that some zoonotic infections can influence the course of psychiatric disorders, especially *Toxoplasma gondii*, *Borrelia burgdorferi *and Borna disease virus (BDV). Borna disease virus (BDV) is an RNA virus belonging to the family Bornaviridae, order Mononegavirales. Borna disease virus affects the central nervous system (CNS), especially limbic structures, and causes infection in animals, including humans and birds. The symptomatology in animals ranges from mild, subclinical infection to lethal meningoencephalitis. This viral infection is associated with neurological, behavioural, mood and cognitive changes [1].

Borna disease virus can cause persistent infection of the CNS. Persistent viral infection is characterised as those circumstances in which the virus is not cleared but remains in the cells of infected individuals. There are three types of persistent viral infection: latent, chronic and slow infection [2]. The latent type of persistent infection is typical for BDV. Latent infection is associated with a lack of demonstrable viral particles. The reactivation of persistent latent BDV infection can be triggered by several stimuli: immune changes (immunosuppression), stress factors, superinfection by other viruses or trauma [1-3].

Borna disease virus infection antigens (Ag), antibodies (Ab), circulating immunocomplexes (CIC) and RNA can be isolated from the brain tissue, cerebrospinal fluid and serum. After activation of latent BDV infection it is possible to detect Ag. In the second phase of acute viremia, Ag binds with Ab and forms CIC. Originally for diagnostics of BDV infection were used detection of viral Ab. The positive finding of BDV Ab means that the organism has been in contact with BDV but it does not necessarily imply an active BDV infection [4,5].

Borna disease virus is transmitted between humans, animals and humans and animals by infected saliva or other secretions through the nasal mucosa. It spreads intra-axonally and trans-synaptically towards olphactoric structures and then to the limbic system. During later infection, BDV diffuses throughout the CNS and can be detected in the peripheral nervous system (astrocytes, Schwann cells, oligodendrocytes).

Borna disease virus causes several changes in brain functions resulting in mood, behaviour, cognitive and neurologic disturbances including movement impairment. During infection, BDV influences the CNS in several ways: firstly a direct influence through binding of viral proteins with neurotransmitter receptors, and secondly an indirect influence through immune response and inflammatory reactions. Both types of mechanisms influence neurotransmission and lead to mood, behaviour and emotional changes in infected individuals and may be associated with human psychiatric disorders (affective disorders, addictions and psychotic disorders). The severity of clinical symptoms of BDV infection depends on the immune response of the host [3,6-8].

Viral infections can influence the human genome. The part of human genetic material originates from viruses and viral sequences assimilate into the host (human) genome. After infection BDV sequences are integrated into the genome of brain cells. These sequences are not heritable but can cause mutations which interfere with brain functions and can contribute to the development of psychiatric disorders [9].

Borna disease virus affects dopaminergic neurotransmission in the central nervous system. These changes support the possibility of a link between BDV infection and human neuropsychiatric disorders which are connected with the impairment of the dopaminergic system, such as schizophrenia, addictions and extrapyramidal disorders [10].

An increased rate of BDV infection occurs in psychiatric patients (with diagnoses of depression, bipolar affective disorder and schizophrenia) [3-5] and immunocompromised patients, especially those with cellular immunosuppression (including HIV-infected patients) [11].

Borna disease virus positivity in psychiatric patients ranges from negative to highly positive (about 90-100%). These differences can be caused by variances in laboratory methods, biological materials, psychiatric diagnosis and the severity of psychopathology in patients and geographical regions.

Detection of BDV antibodies was first achieved by Rott and colleagues in psychiatric patients in 1985 using an IFA method (immunofluorescence assay). There was a demonstrated BDV Ab positivity in 1-4% of psychiatric patients and in 20% of acutely depressed patients [5,12,13]. A higher BDV positivity based on the detection of viral Ag and CIC has been found in psychiatric patients (between 50% and 90% positivity) compared with healthy individuals (between 20% and 30%) by using the ELISA method. The strength and duration of BDV CIC positivity correlated with the severity of symptoms (higher amounts of BDV CIC and Ag were detected in patients with severe psychopathology in comparison with lower levels in patients with mild symptoms) [4,5]. Several studies failed to detect BDV in psychiatric patients and did not confirm the higher positivity [14-18]. It is supposed that BDV infection can modulate the course of psychiatric disorders and play a role in their pathogenesis.

Alcohol and drug abuse alters dopaminergic, serotonergic and nonmonoaminergic systems which lead to mood and behavioural changes [19,20]. Alcohol and drug abusers have an impaired immune system and this impairment is manifested in several ways, including an increased susceptibility to bacterial and viral infections [21-23].

We hypothesised that patients with alcohol and drug dependence would have a higher BDV CIC positivity than healthy individuals as a result of immune changes due to chronic abuse. These immune changes lead to the activation of persistent BDV infection. We expected a higher BDV CIC positivity at the beginning of detoxification (at the time of alcohol and drug abuse) and a reduction after eight weeks of abstinence due to the recovery of the immune system and somatic status.

Our study was approved by the ethical committee of the University Hospital Medical Faculty of Charles University in Pilsen, Czech Republic (registration number 303/2001). The aim and procedures were explained and informed consent was obtained from all participants.

## Methods

### Study population

We examined two groups: patients with alcohol and drug dependence and a control group of healthy individuals (blood donors). Patients included in the study fulfilled the following criteria: 1) the patients were hospitalised in the detoxification unit of the Psychiatric Department of the University Hospital in Pilsen in the Czech Republic and continued short-term treatment of addiction for eight weeks between 2007 and 2008; 2) the patients had a diagnosis of alcohol or drug dependence according to ICD-10 (International Statistical Classification of Diseases); 3) the patients were aged 18 years or older; 4) the patients taking immunosupressive drugs or treated with chemotheraphy and actinotheraphy were excluded from the study. We included 41 patients (18 males, 23 females) of whom 22 had alcohol dependence and 19 had drug dependence, aged from 19 to 62 years (average age 36.29 +/- 12.06 years, median 35 years).

For the control group we examined blood donors (n = 126, 97 males, 29 females, aged from 25 to 65 years, average age 40.31 +/- 11.32 years, median 37 years) from the blood bank of the Central Military Hospital in Prague.

### Laboratory methods

Four millilitres of venous blood was taken from all participants (patients and blood donors). The sera were separated by centrifugation of fresh venous blood within 6 hours after venipuncture. Serum samples were frozen and stored at -80°C until analysed.

**Figure 1:**
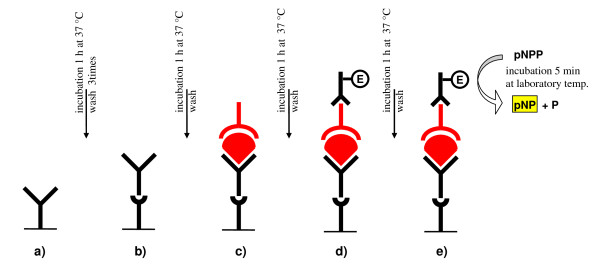
**Detection of BDV circulating immunocomplexes (CIC) in serum**. a) The antibody against mice IgG (against its Fc fragment) is bound at the surface of the microtitrate plate; b) Mice monoclonal antibody against BDV antigens p40 a p24 is added; c) Patient serum is added (in which we are detecting CIC, ie. the complex of BDV antigen and human IgG); d) Goat antihuman IgG (against its Fc fragmen) conjugated with enzyme (ALP - alcaline phosphatase) is added; e) Substrate of ALP *p*-nitrophenylphosphate (pNPP) is added; pNPP is hydrolysed by ALP and released *p*-nitrophenol (pNP) has intensive yellow colour, which is detected by spectrophotometry using microtitrate plate reader (wave length 405 nm).

The BDV-specific CIC in the sera of patients and blood donors were determined by EIA (enzyme immuno assay) using a washing apparatus (MRW Dynex) and an ELISA reader (MUREX MRX). The laboratory method for BDV CIC detection was developed by Bode and Ludwig and uses specific monoclonal antibodies to trap the antigen part of the CIC [4]. The authors of this method provided the monoclonal antibodies W1 and Kfu2 for our study. The antibody part of the CIC was visualised using an enzyme reaction (alkaline phosphatase) (Figure 1). This method was semi-quantitative. Serum samples with a reciprocal titre of 20 (cut off titre 1/20) or more were taken as positive. The levels of BDV CIC were scored as: 0 negative, + low positivity, ++ mean positivity, +++ and ++++ high positivity.

### Study design

All patients were examined for BDV CIC positivity at the beginning of detoxification (day 0) and after an eight-week period of abstinence (day 56). At the same time standard screening laboratory tests (blood tests, liver enzymes, hepatitis A, B and C, tuberculosis, syphilis, human immunodeficiency virus (HIV)) were performed to evaluate the patients' somatic status. Laboratory tests for BDV CIC positivity and liver enzymes were performed on days 0 and 56. A period of 8 weeks is sufficient for the detection of possible changes in levels of BDV CIC and in the values of liver enzymes (especially GGT).

Anamnestic and demographic data (age, gender, psychiatric family history including addictions, personal history of somatic and infectious diseases such as hepatitis and other zoonosis), the duration of abuse (in years), the presence of infection at the time of blood sampling, the diagnosis according to ICD-10 and contact with animals (breeding, farming, pets) were obtained from patients and medical record examination.

### Statistical analysis

Both groups were compared according to age and gender using the Wilcoxon rank-sum test, contigency tables and the chi-square test. The non-parametric Spearman's rank and Kendall tests were used to test for interdependence between variables and the Wilcoxon test was used to compare mean values. Contingency tables and the chi-square test were used in cases where there was a small number of values for the data.

## Results

We examined 41 patients on day 0 and 28 patients on day 56; 13 (31.7%) patients finished their treatment prematurely. The detoxification of addicted patients is often prematurely ended because of eroded abstinence or other treatment regimes. The characteristics of addicted patients are illustrated in Tables 1 and 2.

**Table 1:** Characteristics of addicted patients

Characteristics	Range	Average	Median
Age (years)	19-62	36.29 +/- 12.06	35.0

Duration of abuse (years)	2-15	6.32 +/- 3.60	5.0

GGT day 0 (ukat/l)	0.18-23.07	3.21 +/- 5.586	0.59

GGT day 56 (ukat/l)	0.11-3.13	0.682 +/- 0.755	0.345

**Table 2:** Diagnosis of addicted patients according to ICD-10

Diagnosis	No. of patients (%)	No. of females	No. of males
F 10.2 alcohol dependence	24 (58.5)	14	10

F 11.2 heroin dependence	3 (7.3)	0	3

F 13.2 benzodiazepine dependence	2 (4.9)	2	0

F 15.2 metamphetamine dependence	12 (29.3)	7	5

Age differences between the addicted patients and the control group were statistically significant (p = 0.048, the Wilcoxon rank-sum test). The groups also differed significantly in gender (contingency tables, the chi-square test). No association between BDV CIC positivity and gender was demonstrated (p = 0.884, contingency tables, chi-square test). The difference in BDV CIC positivity was adjusted for age and gender and was not statistically significant (age p = 0.302, gender p = 0.498, higher order contingency tables, the chi-square test).

A BDV CIC positivity was detected in 36.6% of patients (15/41) on day 0 and in 42.9% (12/28) patients after eight weeks of abstinence (day 56) in comparison with a BDV CIC positivity of 37.3% (47/126) in the control group (Table 3). The difference in BDV CIC levels in the serum between these two groups was not statistically significant on day 0 (p = 0.179, contingency tables, the chi-square test) or day 56 (p = 0.223, contingency tables, the chi-square test). There was no reduction in BDV CIC levels over the abstinence period (p = 0.779, contingency tables, goodness of fit test, the chi-square test).

**Table 3:** Levels of BDV CIC in addicted patients on day 0 and 56 and in blood donors

	Psychiatric patients						Blood donors		
**Level of BDV CIC**	**Day 0 (n = 41)**			**Day 56 (n = 28)**			**(n = 126)**		

	**No. (%)**	**No. of females**	**No. of males**	**No. (%)**	**No. of females**	**No. of males**	**No. (%)**	**No. of females**	**No. of males**

Positive	15 (36.6)	8	7	12 (42.9)	6	6	47 (37.3)	10	37

+ (low)	11 (26.8)	6	5	9 (32.1)	3	6	44 (34.9)	9	35

++ (mean)	3 (7.3)	1	2	3 (10.7)	3	0	3 (2.4)	1	2

+++ (high)	0	0	0	0	0	0	0	0	0

++++ (high)	1 (2.4)	1	0	0	0	0	0	0	0

Negative	26 (63.4)	15	11	16 (57.1)	12	4	79 (62.7)	19	60

We monitored changes in BDV CIC levels during the study on days 0 and 56. For patients who provided both blood samples:

• in three patients we detected a decrease in BDV CIC levels (two patients were BDV CIC positive on day 0 and became negative on day 56, one patient's positivity decreased from mean (++) to low (+));

• in six BDV CIC-positive patients we found no change in BDV CIC levels; and

• in five patients we determined an increase of BDV CIC levels (four patients were BDV CIC negative on day 0 and became positive on day 56).

We looked at the association of BDV CIC positivity and other anamnestic and demographic characteristics:

• twenty-two (53.7%) enrolled patients had a positive psychiatric family history;

• five (12.2%) had a positive history of infectious diseases (hepatitis C);

• infection at the time of blood sampling was present in five (12.2%) patients on day 0 and in two (7.1%) patients on day 56 (the presence of another infection can increase the risk of reactivation of latent BDV infection); and

• thirty-seven (90.2%) patients had pets or other animals (animals can be a reservoir of BDV infection and contact with infected animals increases the risk of BDV infection transmission).

We did not establish any other significant association of BDV infection with other anamnestic and demographic data based on contingency tables, the chi-square test and the Kruskal Wallis test on days 0 and 56:

• gender (day 0 p = 0.706, day 56 p = 0.045);

• psychiatric family history (day 0 p = 0.713, day 56 p = 0.241);

• personal history of infectious diseases (day 0 p = 0.692, day 56 p = 0.813);

• presence of infection at the time of sampling (day 0 p = 0.825, day 56 p = 0.452);

• duration of abuse (day 0 p = 0.918, day 56 p = 0.436);

• diagnosis according to ICD-10 (day 0 p = 0.468, day 56 p = 0.557); and

• contact with animals (day 0 p = 0.611, day 56 p = 0.446).

A higher BDV CIC positivity was significantly associated with lower levels of GGT (gamma-glutamyl transferase) at the beginning of detoxification (p = 0.027, rank correlation with the Kruskal Wallis test) and approached statistical significance with the lower age of addicted patients (p = 0.064, rank correlation with the Kruskal Wallis test).

We proved no association between BDV CIC positivity on day 0 and premature ending of the treatment. Premature ending of the treatment was not associated with BDV CIC positivity on day 0 (p = 0.645, contingency tables, the chi-square test).

## Discussion

In this study the positivity of BDV CIC was detected in 36.6% of addicted patients at the beginning of detoxification, in 42.9% after eight weeks of abstinence and in 37.3% of the control group of blood donors.

Surprisingly, we did not find a higher BDV CIC positivity in patients with alcohol and drug dependence in comparison with healthy individuals. Nor did we find any significant difference between BDV CIC positivity at the beginning of detoxification and after eight weeks of abstinence. A BDV CIC positivity was not associated with the premature ending of treatment. We cannot compare our results with other studies because no other studies of BDV infection in addicted patients have been published. Larger studies are needed to confirm these results.

We assumed a higher positivity of BDV infection in addicted patients because of the higher BDV CIC positivity found in psychiatric patients in the Czech Republic, and possible changes in immunity and close relationships with endemic regions for BDV in central Europe. The positivity of BDV CIC in addicted patients in the present study was lower than that found in our previous study in psychiatric patients (with psychotic and affective disorders). We detected BDV CIC positivity in 66.7% of psychiatric patients and the intensity of BDV infection was positively correlated with psychopathology [24].

Bode and colleagues detected a BDV CIC positivity of between 40% and 50% in psychiatric patients and between 90% and 100% in patients with an acute state of affective disorders in comparison with a positivity of 30% in blood donors. The persistence of high amounts of plasma BDV CIC and Ag correlated with the severity of depression [4].

Nunes and colleagues examined BDV RNA by using reverse transcriptase polymerase chain reaction in psychiatric patients, their relatives and healthy controls. Borna disease virus RNA positivity was detected in patients with psychotic disorders (44.4%), relatives without mental disorders (50%), relatives with mental disorders (37.5%) and healthy controls (14.8%) [25].

Another study failed to detect BDV positivity in psychiatric patients (depression, bipolar disorders, schizophrenia) where neither the BDV antibody nor the BDV RNA was proven in psychiatric patients or healthy controls [18]. The authors of this study suggested that BDV infection might not be associated with mental disorders in this region.

We examined the presence of BDV CIC in the serum by using the ELISA method and were able to detect active BDV infection. The ELISA method finds a higher BDV positivity than other laboratory methods (IFA) [4,5].

Borna disease virus infection (antibodies) was first detected using serological methods, especially immunofluorescence (IFA), by Rott and colleagues [13]. A positivity of BDV Ab was found of between 1% and 4%. Another laboratory method which has been used for the detection of BDV Ab is western blot (WB). These methods detecting only BDV Ab were found to be less sensitive than the ELISA method and were not able to detect acute phases of BDV infection. Antibodies, when found alone, can indicate a previous contact with BDV but not an acute state. Bode and colleagues examined plasma samples using ELISA for BDV CIC positivity and immunofluorescence for the detection of antibodies. The BDV CIC determination found a higher prevalence of infection than previous IFA methods (ten times higher infection rates) [4,26].

Antigenaemia indicates an acute and productive phase of infection. During this phase of BDV infection antibodies bind to the antigens and form circulating immunocomplexes (CIC), which are measurable for weeks or months. The frequency and stability of BDV CIC makes them available screening markers of BDV infection, as recommended by some authors. By using the ELISA method it is possible to detect viral Ag in plasma. A higher positivity of BDV Ag was detected in patients with depression and the intensity and duration of antigenaemia was correlated with the severity of symptoms [4,5]. The disadvantage of this detection method is the very short period of antigaenemia in the acute phase of BDV infection [4,5,26].

Several authors used the detection of viral RNA in PBMCs (peripheral blood mononuclear cells) or brain tissue by polymerase chain reaction (PCR) for the diagnosis of BDV infection. However, other researchers did not use this method for the diagnosis of BDV infection because of the possibility of sample contamination during the laboratory procedures [26], although this contamination should not occur when the detection of BDV RNA is performed according to international security instructions [25]. The absence of BDV RNA in the serum cannot exclude the presence of infection as the low amount of RNA is not possible to detect using this method. The second reason for not using BDV RNA detection is that that the presence of BDV RNA does not reflect an active state of viral replication [26].

A limitation of our study was that only BDV CIC positivity was detected. Antigens and circulating immunocomplexes are the markers of BDV infection activity. Some CIC-negative patients can have acute viraemia and are only Ag positive. Furthermore, the immune impairment in addicted patients lowers the ability to produce Ab and provides far less CIC formation. Also, immune changes in addicts are not important for BDV infection.

We found a higher BDV CIC positivity in younger patients and a decrease in BDV CIC positivity with the increasing age of patients. This finding is consistent with the observations of the Polish psychiatric population [27] and German [28] and Italian [29,30] studies which demonstrated a significantly higher BDV CIC positivity in children. Patti and colleagues investigated BDV positivity in children in Italy. A BDV CIC positivity was found in 57%. The prevalence of BDV infection was found to be higher in children, particularly in the third year of life, then, it decreased until 15 years of age, where another increase was observed [29,30]. Scholbach and colleagues demonstrated a higher BDV Ag and CIC positivity in children. There were two age intervals of peak BDV positivity, the first with a peak at 6 months old and the second with a peak value around 2-3 years old. These findings supported the possibility of vertical transmission of BDV infection. Also, children of 2-3 years old are likely to be in more intensive contact with the secretions of animals, which are associated with a greater possibility of BDV transmission in children than in adults [28].

Flower and colleagues found a positive association between BDV positivity and elevated liver enzymes. They described the association between BDV antigenaemia and elevated plasma levels of ALT and GGT in multi-transfused patients. It was not clearly explained whether these findings had any causal associations [31]. In our study we detected the opposite situation, where a higher BDV CIC positivity was significantly associated with a lower level of GGT, implying milder liver impairment. We are not able to satisfactorily explain this finding yet. The influence of a lower age (i.e. a shorter history of abuse and/or higher frequency of non-alcoholic drug abuse) was not confirmed.

Alcohol and drug abuse interferes with humoral and cellular immunity and leads to the suppression of human resistance to bacterial and viral infections, increasing infection susceptibility. This direct influence on immunity in combination with other risk factors (risk behaviour, vitamine deficiency) is associated with impairment of the immune system [22,23].

We proved no significant association between BDV CIC positivity and the history of infectious diseases (hepatitis C) or the presence of infection at the time of blood sampling. The presence of other infections can reactivate the latent BDV infection and cause the increase of BDV positivity [2,3]. Cotto and colleagues investigated the presence of BDV infection (BDV RNA) in two groups of immunocompromised patients (with HIV infection and treated with immunosuppressive medication). They detected a significantly higher BDV positivity in patients with HIV infection compared with the second group and healthy controls [11].

In our study, contact with animals was not associated with BDV CIC positivity, although the majority of the addicted patients from our study had been exposed to animals (especially pets such as dogs and cats).

Some studies described a higher BDV positivity in people who were in contact with infected animals. Weisman and colleagues reported a significantly higher positivity of BDV antibodies (46%) in workers exposed to infected ostriches versus a BDV positivity of 10% in controls. There was a strong correlation between the intensity of exposure and the rate of seropositivity [32]. Takahashi and colleagues found a significantly higher seropositivity of BDV (from 2.6% to 14.8%) in blood donors from regions where horse farms were concentrated compared with a BDV positivity of 1% in blood donors from other regions [33]. These findings support the possible animal-to-human transmission of BDV infection. In contrast, another study from Bangladesh did not confirm this hypothesis. The authors surveyed horses and their caretakers for BDV antibody positivity and found a BDV positivity of between 25-30% in the horses but none of caretakers were positive for BDV [34]. Thomas and colleagues reported that people working or living on livestock farms had a higher BDV seroprevalence compared with other farms. Exposure to animals did not increase the risk of BDV positivity [35].

Another explanation for not finding an association between contact with animals and BDV CIC positivity is that these results might have been derived from an area in which the BDV infection of animals is not endemic. We still have no data about the prevalence of BDV infection in animals in the Czech Republic. Several studies found a high BDV positivity in horses and sheep [1,36] and a lower BDV positivity in other species (dogs, cats, cattle) [1]. Addicted patients from our study were frequently exposed to pets such as cats and dogs (90.2%) but they were not exposed to farm animals, which we could expect to have a higher BDV positivity than pets.

## Conclusions

This study compared BDV CIC positivity in addicted patients and healthy individuals. We detected no significant difference in BDV positivity between these two groups and between BDV positivity on days 0 and 56. We found an association between BDV infection and the levels of GGT and the age of the patient. Additional studies will continue to examine BDV CIC and Ag in psychiatric inpatients and addicted patients.

## List of abbreviations

Ag: antigen; Ab: antibody; BDV: Borna disease virus; CIC: circulating immunocomplexes; CNS: central nervous system; GGT: gamma-glutamyl transferase; HIV: human immunodeficiency virus; EIA: enzyme immunoassay; ELISA: enzyme-linked immunosorbent assay; IFA: immunofluorescence assay; ICD-10: International Statistical Classification of Diseases; PCR: polymerase chain reaction; RNA: ribonucleic acid;

## Competing interests

The authors declare that they have no competing interests.

## Authors' contributions

SR designed the study protocol, obtained the approval of the local ethical committee, wrote the manuscript, performed the clinical evaluation of psychiatric patients and performed the evaluation of psychiatric patients with psychiatric scales and anamnestic questionnaires and the blood sampling for the BDV CIC examination.

LJ designed the study protocol, wrote the manuscript and performed the clinical evaluation of psychiatric patients and the evaluation of psychiatric patients with psychiatric scales and anamnestic questionnaires.

HK performed the laboratory tests on blood samples from psychiatric patients and blood donors for BDV CIC positivity.

All authors read and approved the final manuscript.

## Pre-publication history

The pre-publication history for this paper can be accessed here:

http://www.biomedcentral.com/1471-244X/10/70/prepub
